# Barriers and facilitators to self-measured blood pressure monitoring among US-resettled Arab refugees with hypertension: a qualitative study

**DOI:** 10.1186/s12875-023-02215-1

**Published:** 2023-11-30

**Authors:** Lana Bridi, Behnan Albahsahli, Nissma Bencheikh, Dania Abu Baker, Job G. Godino, Kelli N. O’Laughlin, Tala Al-Rousan

**Affiliations:** 1 School of Medicine, University of California, San Diego, San Diego, CA USA; 2 Herbert Wertheim School of Public Health and Human Longevity Science, University of California, San Diego, San Diego, CA USA; 3 School of Social Work, San Diego State University, San Diego, CA USA; 4 Laura Rodriguez Research Institute, Family Health Centers of San Diego, San Diego, CA USA; 5 Departments of Emergency Medicine and Global Health, University of Washington, Seattle, WA USA

**Keywords:** SMBP, Home blood pressure monitoring, Self-management, Refugee health, Non-communicable disease, Chronic disease, Theory of care-seeking behavior

## Abstract

**Background:**

Minoritized communities including refugees are at an increased risk of poorly controlled hypertension. Evidence indicates that self-measured blood pressure monitoring (SMBP) is an effective method to improve blood pressure control in patients with hypertension. However, it has not been studied among refugee populations. The objective of this study is to examine barriers and facilitators to SMBP among Arab refugees resettled in the United States (US) with diagnosed hypertension.

**Methods:**

A total of 109 participants were recruited through a Federally Qualified Health Center system that is a major provider of healthcare to refugees in San Diego, California. Participants completed a questionnaire and were interviewed using in-depth, semi-structured interviews. Interviews were transcribed and translated, and data were coded using inductive thematic analysis and organized based on the theory of care-seeking behavior.

**Results:**

Several barriers to engaging in effective SMBP monitoring were identified. Clinical and sociodemographic barriers included reliance on public monitors and poor hypertension literacy. Psychosocial barriers of affect, norms, and habits included fear and anxiety from hypertension, cultural stigma of illness, and conditional SMBP with symptoms, respectively. Utility psychosocial barriers included lack of SMBP prioritization in treatment and perceived inaccuracy of home monitors. Family members’ support with home monitoring served as an important facilitator to SMBP.

**Conclusions:**

There are several barriers to effective SMBP among the US-resettled Arab refugee population that may reflect unique cultural and care-seeking behaviors. Tailored public health and clinical interventions are needed to support refugee patients and providers to improve hypertension self-management behaviors for this unique population.

## Background

Hypertension is one of the leading preventable risk factors for all-cause mortality and one of the most important modifiable risk factors for coronary heart disease and stroke [1–3]. Globally, hypertension is a significant public health burden, causing an estimated 7.5 million deaths annually, around 12.8% of all deaths, and 57 million disability-adjusted life years [4]. Despite targeted health efforts, rates of hypertension and hypertension-related mortality have been increasing, and rates of blood pressure control have been declining (5–6). In the United States (US), the proportion of people with controlled hypertension – defined as systolic blood pressure < 140 mm Hg and diastolic blood pressure < 90 mm Hg – declined from 51.3% in 2013–2016 to 48.2% in 2017–2020 [7].

Racial and ethnic disparities in hypertension rates and outcomes in the US have been well documented in the literature, however, such studies rarely analyze Arab Americans as a distinct racial/ethnic group of interest [8–10]. Very little research has focused on hypertension among Arab Americans. Of that research, a cross-sectional study using data collected over 10 years ago has shown that blood pressure control is lower among Arab Americans compared to national rates [11]. Although hypertension rates and blood pressure control are not as well-studied among refugee populations, some research has demonstrated increased burden of cardiovascular disease among refugees, with uncontrolled blood pressure being the most prominent risk factor [12–14]. A scoping review of the literature revealed that adult refugees – including Arab refugees from Iraq and Syria – are at higher odds of having non-communicable diseases such as hypertension than their non-refugee counterparts [15] and poor hypertension control [16–19].

Risk for cardiovascular disease increases with even small increases in blood pressure [20], thus, establishing effective regimens for hypertension control is a significant public health priority. Self-measured blood pressure monitoring (SMBP), also known as home blood pressure monitoring, is increasingly used in many countries for its potential to improve self-management of hypertension [21]. Guidelines from the American Heart Association have endorsed the use of SMBP in clinical practice as a useful supplement or alternative to conventional office measurements [22]. The benefits of SMBP include providing longitudinal data on blood pressure; it also predicts risk of cardiovascular events and target organ damage better than conventional office measurements and can detect white coat and masked hypertension phenomena (21–22).

Multiple studies have demonstrated SMBP leads to therapeutic compliance and improved blood pressure control [22–25]. For example, a randomized controlled trial testing a digital SMBP intervention revealed a mean blood pressure drop from 151.7/86.4 to 138.4/80.2 mm Hg after one year, giving a mean difference in systolic blood pressure of -3.4 mm Hg compared to the usual care group [23]. Other studies demonstrated SMBP facilitates interactions with providers and patient engagement in their own care (26–27). Although these studies exhibit clear benefits of SMBP, the overwhelming majority of the literature has focused only on White patients with no migration history. One study from the US attempted to address this literature gap by investigating the effectiveness of SMBP in underserved populations, predominantly Black and Hispanic; they found that providing a home blood pressure monitor did not improve control over usual care, highlighting additional barriers to blood pressure control that need to be studied [28]. There are currently no studies on SMBP among refugee populations, despite a demonstrated need for research on self-management practices among refugees with chronic diseases [29].

With increasing forced displacement and resettlement of refugees in the US, it is imperative to understand this population’s chronic disease self-managements, particularly among refugees from Iraq and Syria since the Centers of Disease Control and Prevention list hypertension as a priority health condition for these populations (30–31). Due to years of political and economic unrest, many refugees have been displaced from the Middle East; 9.2 million Iraqis have been displaced as of 2021, and 13.5 million Syrians have been displaced, representing more than half of Syria’s total population (32–33). San Diego, California (CA) is one of the largest US resettlement cities, with immigrants and refugees accounting for 21.5% of the population (34–35). Since the 1980s, San Diego County has resettled many Iraqi refugees due to the availability of multiple resettlement agencies. This made the area one of the largest US homes to the Iraqi diaspora and rich with Arabic resources for displaced people, which later supported the resettlement of many Syrian refugees [31, 34, 36]. This study aims to document San Diego Arab refugees’ experience with SMBP and to close the literature gap on refugee health experiences with this method for hypertension self-management. Specifically, this qualitative study assesses the barriers and facilitators to engaging in regular SMBP within the US-resettled Arab refugee population.

## Methods

### Design

This study is part of a larger investigation into refugee patients’ barriers to hypertension care and opportunities to improve self-management (IRB #200,063). The investigation was comprised of three sequential parts: (1) an initial interview with participants regarding their hypertension and self-management; (2) collection of home blood pressure readings after participants were provided a free Withings BPM Connect, an FDA-approved, cellular-connected home blood pressure monitor, and were instructed to maintain a blood pressure diary; (3) a follow-up interview regarding participants’ experience with the home monitoring. Our current study stems from part one of our larger investigation. Here, we employed an exploratory qualitative investigation into the beliefs, attitudes, and behaviors that facilitate or prevent SMPB among Arab refugees with hypertension. Applied thematic analysis was used in this study because of its inductive procedure that presents participants’ experiences as comprehensively and accurately as possible [37]. This study followed the Standards for Reporting Qualitative Research (SRQR) [38].

### Participants

Participants were Syrian and Iraqi refugee patients with a diagnosis of hypertension at the Family Health Centers of San Diego (FHCSD), a Federally Qualified Health Center system that is a major provider for refugees in San Diego, CA. Inclusion criteria were: (1) having been diagnosed with hypertension (i.e., prescribed at least one antihypertensive medication, confirmed through the FHCSD electronic health record); (2) having a present or former refugee status; (3) being from Iraq or Syria; (4) living in San Diego for 3 months after recruitment date. Exclusion criteria were: (1) anyone under 21 years old; (2) those unable to provide informed consent.

Recruitment occurred between April 2021 and April 2022. Our native Arabic-speaking investigators, including authors LB and BA, were trained to follow a standard operating procedure for recruitment and consenting processes in Arabic. They randomly selected potential participants to contact via phone from a list of eligible patients procured by the FHCSD. Interested participants were screened for inclusion and exclusion criteria over the phone and consented. A total of 109 participants completed this study.

### Data collection

Data was collected through semi-structured, in-depth interviews and a questionnaire for demographics data [39]. All investigators involved in data collection, including authors LB, BA, and TA, were Middle Eastern native Arabic-speakers. LB and TA were previously trained in qualitative methods and TA is a clinical professional. Data was collected either through an in-person interview or a virtual interview based on the current local COVID-19 pandemic safety protocols and the participant’s preference. In-person interviews were completed at the Majdal Community Center, an ethnic-based community organization that works directly with the target population. Virtual interviews were conducted on Zoom’s password-protected video-conferencing platform. The interview guide was developed by research experts and clinical providers and was informed by a scoping review of the literature (Table 1). Interviews explored current SMBP habits and factors that facilitate or prevent SMBP. Interviews ranged from 30 to 90 min and were conducted by trained bilingual Arabic-speaking investigators on Zoom or in-person at the Majdal Community Center. For safety precautions, interviewers were trained in referring participants who exhibited safety concerns or suicidal thoughts to designated clinics serving refugees locally. Additionally, all in-person interviews followed current local COVID-19 safety guidelines. Interviews were audio-recorded, transcribed in Arabic, translated into English, and reviewed for content and accuracy.

**Table 1:** In-depth interview guide questions selected for qualitative data analysis

Domain of Inquiry	Questions
Attitudes and knowledge of hypertension	Does high blood pressure worry you? Tell me why. What do you think might happen as a complication from high blood pressure? What would make life better for people with high blood pressure? Any final thoughts for what you think may achieve good blood pressure control?
Current SMBP behaviors	Do you get your blood pressure regularly checked? (probe for public device use, availability of home monitor)
Barriers and facilitators to SMBP	Do you face difficulties in getting your blood pressure checked regularly? What are they? Do you think your monitor is accurate? Why? In what ways has the refugee experience impacted your hypertension management?

### Data analysis

Inductive thematic analysis was used to analyze the translated transcripts. Investigator triangulation was implemented by authors LB, BA, DAB, and NB who completed the analysis. LB and DAB trained BA and NB on qualitative analysis techniques. Using ATLAS.ti software, LB and BA independently reviewed and open coded a set of transcripts (n = 5) and met regularly to establish a codebook through an iterative process. The coders used the subjective assessment method to establish intercoder agreements [37]. Analysis was done by the above four authors through an iterative process identifying recurrent themes following Crabtree and Miller’s 5-step interpretive process: describing, organizing, connecting, corroborating/legitimating, and representing the account [40]. Transcripts were analyzed until inductive thematic saturation was reached, defined as the process in which all the coders collectively agreed that additional data did not lead to new emergent themes [41]. A total of 54 transcripts were analyzed.

### Trustworthiness of data

Our study involved the following strategies to ensure trustworthiness: prolonged engagement, investigator triangulation, and thick description [42]. We achieved prolonged engagement through investing significant time in the field to understand participants’ access to healthcare resources and establish community partnerships, our one-year duration of this study, our large number of interviews, and follow-up probing during interviews. We achieved investigator triangulation as described above. These two methods enhance our study’s credibility. Transferability of the data is ensured by thick descriptions detailing this study’s methodology, participant sample, focus group guide, and qualitative findings.

### Ethical approval

Informed consent was obtained by all participants. Participants had the right to withdraw from the study without penalty or loss of benefits to which they are entitled, and their data would be destroyed and not included in this study. The Institutional Review Board (IRB) at the University of California, San Diego approved this research (#200,063).

## Theoretical framework

This study adopted the theory of care-seeking behavior developed by Lauver to explore factors that influence Arab refugees’ SMBP practices [43]. This theory was developed from Triandis’ earlier theory of behavior [44]. The theory of care-seeking behavior has been heavily applied to the field of healthcare utilization among specific groups of people [45]. This theory describes the probability of an individual to engage in health behaviors as a function of psychosocial variables – including affect, utility, norms, and habits – and facilitating conditions.

Affect refers to emotions associated with care seeking including fear of a diagnosis or treatment. Utility encompasses the expectations and values about a certain outcome, reflecting the individual’s perception of the overall worth of care seeking. Norms include both social norms and personal norms. Habits refer to how an individual typically reacts to symptoms. Facilitating conditions are specific external variables that enable an individual to engage in care-seeking behaviors, for example, access to medications.

The theory of care-seeking behavior claims that psychosocial variables can influence behavior either directly or in interaction with facilitating conditions. It also acknowledges that variables extrinsic to the theory such as clinical and sociodemographic factors can influence behavior, however, only through the identified psychosocial variables.

This study used the theory of care-seeking behavior to organize the themes extracted from participant interviews to exhibit the interrelationships between the themes and their influence on SMBP.

## Results

**Fig. 1:**
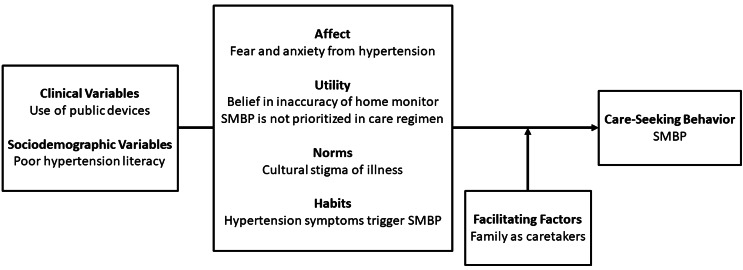
Care-seeking behavior theory applied to Arab refugees and SMBP based on the qualitative data collected in this study. This figure, adapted from Lauver’s original [43], depicts how the probability of engaging in SMBP for Arab refugees is a function of their clinical and socio-demographic variables, psychosocial variables (affect, utility, norms, and habits), and facilitating conditions

Data on demographics and blood pressure monitoring practices of participants is provided in Table 2. The interviews from this study yielded eight themes on factors that either facilitate or prevent SMBP among Arab refugees. (1) SMBP through public devices presents barriers (2) Poor hypertension literacy hinders SMBP (3) Fear and anxiety from hypertension impact monitoring (4) Belief in inaccuracy of home monitor discourages its use (5) SMBP is not a priority in hypertension care regimens (6) Cultural stigma of illness as a barrier to SMBP (7) Hypertension symptoms trigger SMBP (8) Family members’ roles as caretakers in SMBP. The themes are organized into the variables described in the theory of care-seeking behavior (Fig. 1) [43].

**Table 2:** Participant demographics and blood pressure monitoring practices

		Iraqi *n =* 85	Syrian *n =* 24	All *n = 109*
Age, years (mean, SD)		63 (9.2)	53 (7.6)	61 (9.7)
Years in the U.S (mean, SD)		10.6 (6.2)	6.0 (1.3)	9.6 (5.8)
Gender				
Female		44 (51.7)	9 (37.5)	53 (48.6)
Male		41 (48.3)	15 (62.5)	56 (51.4)
Marital Status				
Married		70 (82.4)	22 (91.7)	92 (84.4)
Divorced		3 (3.5)	0	3 (2.75)
Widowed		11 (12.9)	2 (8.3)	13 (11.9)
Never Married		1 (1.2)	0	1 (0.95)
Highest level of education				
Less than high school		29 (34.1)	13 (54.1)	42 (38.5)
High school		18 (21.2)	9 (37.5)	27 (24.7)
Vocational certificate		15 (17.6)	1 (4.2)	16 (14.7)
Undergraduate		18 (21.2)	0	19 (17.5)
Postgraduate		5 (5.9)	1 (4.2)	5 (4.6)
Employed				
Yes		13 (15.3)	2 (8.3)	15 (13.8)
No		72 (84.7)	21 (87.5)	93 (85.3)
No Response		0	1 (4.2)	1 (0.9)
Annual income				
Less than $15,000		49 (57.6)	18 (75.0)	67 (61.5)
$15,001 - $25,000		26 (30.6)	5 (20.8)	31 (28.5)
$25,001 - $35,000		7 (8.2)	0	7 (6.4)
$35,001 - $50,000		2 (2.4)	0	2 (1.8)
$50,000+		1 (1.2)	1 (4.2)	2 (1.8)
Proficient in English				
Yes		33 (38.8)	5 (20.8)	38 (34.9)
No		52 (61.2)	19 (79.2)	71 (65.1)
Do you think you have hypertension?				
Yes		77 (90.6)	14 (58.3)	91 (83.5)
No		4 (4.7)	4 (16.7)	8 (7.3)
I don’t know		5 (5.9)	5 (20.8)	10 (9.2)
Can you give us a value or a number if we asked you about your own blood pressure?				
Yes		67 (77.9)	18 (75.0)	85 (78.0)
No		16 (18.6)	4 (17.4)	20 (18.3)
I don’t know		3 (3.5)	2 (8.7)	5 (4.6)
When was the last time you had your blood pressure checked?				
Less than a month ago		72 (84.7)	21 (87.5)	93 (85.3)
1–6 months ago		10 (11.6)	2 (8.7)	12 (11.0)
6–12 months ago		3 (3.5)	1 (4.3)	4 (3.7)

### Clinical factors

#### SMBP through public devices presents barriers

Some participants shared that they do not own or have access to a blood pressure monitor at home. To overcome this barrier, they would access the public blood pressure monitor available at their local pharmacies. Although public devices were able to facilitate SMBP for some participants, others shared that measuring through public devices was not as feasible:

*There are always long waiting lines [at the pharmacy] and I feel like my blood pressure increases more just by waiting in line. (Male, 56)*.

*There is no privacy when measuring your blood pressure at the pharmacy; everyone is staring at your reading. (Female, 52)*.

Importantly, some participants shared how the COVID-19 pandemic impacted their public device use and SMBP:

*Sometimes I used to go to [a pharmacy] and use their public device. After the pandemic, they stopped so I had to stop. (Male, 48)*.

*Before the pandemic, I used to use the pharmacy’s device to measure my blood pressure, but now I bought a device. (Male, 63)*.

### Sociodemographic factors

#### Poor hypertension literacy hinders SMBP

Many participants revealed that they do not know how to engage in SMBP and/or interpret their blood pressure readings. For participants who are motivated to monitor their hypertension through SMBP, most overcome this barrier by relying on family members and friends with stronger health literacy to aid them:

*My daughter is the one that helps me and measures [my blood pressure] for me. I do not know how to use the device. (Female, 53)*.

*I genuinely do not know how to use [my home device] and turn it on. I need to learn how. I am being transparent with you because I really want to learn since I can’t keep relying on my son to come and help me with it, he has his own busy life and schedule. (Female, 57)*.

For participants who depend on their social networks for overcoming this barrier to SMBP, some acknowledged the frustration in being unable to immediately measure their blood pressure when urgently needed:

*My neighbor helps me out when I need to measure my blood pressure since I do not know how to read the measurements… I do not measure it if she is not home to help me. [This bothers me] because [hypertension] is serious. I need to know when my blood pressure is high in case I need to be hospitalized as mentioned by my doctor. (Female, 49)*.

### Affect

#### Fear and anxiety from hypertension impact monitoring

A majority of participants shared that hypertension, and its consequences, are a source of worry. Most were specifically concerned about cardiovascular disease, hospitalizations, and death. Additionally, some of these worries were particularly concerned about the disease’s impact on family:

*[Hypertension does worry me], of course. I heard that hypertension can cause heart attacks and other diseases. Therefore, I always measure my blood pressure and make sure I am managing it. And recently I have developed anxiety and I talked to my doctor about it. This anxiety stems from my fear of something happening to me and leaving my kids on their own. (Male, 47)*.

These fears appear to be heightened among participants with a family history of hypertension or cardiovascular disease. Although this fear associated with hypertension is a negative affect, for some participants, this motivates SMBP:

*Yes, [hypertension] worries me a lot, especially when I go to the doctor’s appointment and I have already taken my medication, eaten well and still see that my blood pressure is high. It really surprises me. Then I go home and check it and it is still high. I keep checking and after 24 hours, it is still high. (Male, 51)*.

For others, it can have the opposite effect:

*When I see that my blood pressure is high, this triggers my anxiety and then this anxiety causes my blood pressure to increase… So, I try my best not to increase my blood pressure, thus, I decided not to measure my blood pressure regularly. I get so scared to see a high reading, so I avoid measuring. (Female, 62)*.

This SMBP-associated anxiety can alternatively manifest as compulsive monitoring behaviors which can serve as a barrier to SMBP:

*Usually, [I measure my blood pressure] once a month or once every three weeks, but when I see it is higher, I start drinking a lot of water and drinking lemons. After one hour, I measure it… or every two to three hours until it decreases all the way to normal. (Male, 51)*.

On the contrary, a few participants were not worried about their hypertension, did not believe that they have hypertension, or did not believe in the disease itself. For example, one participant described their understanding of the disease:

*I heard and read a lot about the disease and how people manage it; I have a friend who was diagnosed with hypertension, yet she never believed it and felt fine. I heard about how pharmaceuticals capitalize on hypertension medications and emphasize the selling of medications. So, I guess with all these conflicting ideas my worry about hypertension fluctuates if it ever existed in the first place. I even do my own research and I never find a solid answer about how to manage hypertension. Same thing goes for the discrepancies in doctors’ opinions. Every time a different doctor gives a different opinion about hypertension… I am not convinced with the disease. (Male, 55)*.

### Utility

#### Belief in inaccuracy of home monitor discourages its use

Most participants interpreted the variability in SMBP readings as a sign that the device is not accurate:

*Sometimes [my home device is inaccurate]. When I measure three times, I get different readings. (Female, 52)*.

*I am not fully convinced with the accuracy of my home device. However, when I am at the clinic and measuring my blood pressure over there, I always find discrepancies between my clinic readings and home readings. Usually, the clinic’s device gives higher readings. (Male, 46)*.

A few participants attributed this variability in blood pressure measurements to a stressful clinical space:

*There is a difference between the clinic’s reading and home reading. At the clinic, [my blood pressure] is usually higher, so I don’t know. Maybe it is because I get more anxious at the clinic. (Male, 46)*.

The belief in the inaccuracy of their home devices serves as a barrier to SMBP:

*I do not [monitor my blood pressure] regularly… As I told you my home device is old, maybe with a new device I will start measuring more regularly. (Female, 51)*.

Others implemented different methods to circumvent the perceived inaccuracy of their home monitors:

*No [I do not measure regularly], only every two months because I have to go to the pharmacy to measure it. My home device is not accurate. (Male, 80)*.

*At this point, I know that my device is within two degrees of error from the clinic’s device, however, I still measure my blood pressure using my device and then I estimate my actual blood pressure by subtracting two points from my device’s reading. (Male, 36)*.

#### SMBP is not a priority in hypertension care regimens

Many participants do not find significant value from engaging in regular SMBP for their hypertension management. Compared to medication regimens or visits with providers, monitoring blood pressure at home is not as prioritized:

*I am not that invested in my blood pressure. I am already preoccupied with my diabetes and insulin shots. (Female, 57)*.

*I used to measure my blood pressure regularly, but when I started taking my medications, I stopped and started measuring my blood pressure every 15–20 days… [I used to keep a journal for my blood pressure] for about a month… Now I do not really [worry about checking my blood pressure]. (Male, 56)*.

*Whenever I am measuring [my blood pressure], it is mostly for reassurance. I count more on my medications [for my hypertension management]. (Male, 49)*.

One reason for the low prioritization of home monitoring is a lack of encouragement from providers. When a participant revealed they did not measure their blood pressure regularly, investigators probed if their provider discussed their blood pressure monitoring:

*I was never [prescribed a device]. My husband had open heart surgery so that is why he was given one. [My doctor did not advise me to measure my blood pressure regularly]. (Female, 51)*.

Others shared similar experiences:

*If my doctor tells me to measure my blood pressure I would do it, however my doctor does not tell me, and I feel normal. If I ever feel dizzy or have any other symptoms, I will definitely measure my blood pressure. (Male, 73)*.

### Norms

#### Cultural stigma of illness as a barrier to SMBP

Some participants cited Arab cultural norms and its stigmatization of illness as a barrier to engaging in SMBP. Participants shared how they feel a social pressure to hide their illness and, consequently, neglect their health:

*Culturally as Arabs, we refuse to admit that we are sick, so we tend to neglect our health. (Female, 40)*.

*The fear comes from the cultural and social stigma perpetuated by our family members and friends. They might make you more anxious about your health and well-being by trivializing the importance of keeping up with one’s own health. I can commit to this intervention and the next day I will find my family and friends questioning my health and telling me undesirable jokes about being fixated with my own health. (Male, 46)*.

This stigma of illness from Arab culture impacts even close familial relationships:

*The idea of taking my blood pressure in front of my kids and showing them that I am sick makes me feel uncomfortable. (Male, 56)*.

### Habits

#### Hypertension symptoms trigger SMBP

Many participants described how they do not have an established SMBP routine. They only engage in SMBP when they experience symptoms they associate with hypertension, for example, headaches and dizziness:

*When I feel frustrated, or heating up, or agitated, I know that my blood pressure is high. This immediately prompts me to measure my blood pressure and take medications to manage it. (Male, 47)*.

*When [I sense my blood pressure] gets high, I start measuring it every three minutes and more frequently. (Male, 68)*.

This conditional SMBP appeared to increase in frequency proportionally to the severity of symptoms:

*I do not [monitor my blood pressure regularly], just when I feel extra tired. Sometimes I check three to four times a day on the days when my symptoms are extreme. (Female, 62)*.

For participants who do not engage in SMBP on a regular schedule, if they do not experience any hypertension symptoms, they are not motivated to monitor their blood pressure:

*When I feel better, I tend to forget to measure my blood pressure. (Male, 66)*.

### Facilitating factors

#### Family members’ roles as caretakers in SMBP

Participants shared how their family members play a role in their hypertension management as caretakers, providing help with blood pressure monitoring and medication adherence:

*Yes, [I need help measuring my blood pressure]. [My husband and I] alternate in helping each other measure our blood pressure. (Female, 63)*.

*When I get migraines, I ask my husband to measure my blood pressure at home. This prompts him to give me my hypertension medications. (Female, 51)*.

*My wife [reminds me to measure my blood pressure]. We also use the same device. (Male, 65)*.

Some participants revealed how encouragement from their loved ones results in SMBP:

*I do not [measure my blood pressure regularly], only when my wife pressures me to measure it. (Male, 55)*.

## Discussion

This study qualitatively examined barriers and facilitators to SMBP among Arab refugees who have resettled in the US. Analysis of the data yielded eight pertinent themes that were classified according to Lauver’s theory of care-seeking behavior [43]. Clinical and sociodemographic variables that impact SMBP included reliance on public monitors and poor hypertension literacy. Psychosocial factors included fear and anxiety from hypertension, belief in the inaccuracy of home monitors, the lack of prioritization of SMBP, stigma of illness, and conditional SMBP with symptoms. Facilitating factors included support from family members.

From the themes on clinical and sociodemographic variables, it is evident that refugees might not have access to the tools needed to engage in SMBP (i.e., a home blood pressure monitor and good hypertension literacy). It is clear from this study’s demographics data – 61.5% have an annual income of less than $15,000 – and previous research that refugees settled in the US face many socioeconomic challenges, therefore securing monitors can be very challenging (Table 2) [46]. However, as Yi et al. have demonstrated in their research, accessing a home monitor does not guarantee SMBP behaviors in medically underserved communities [28]. For this population, poor hypertension literacy is an important barrier to SMBP that would not be alleviated simply by having access to a home monitor. These results are consistent with other studies that have examined medication adherence among refugees with hypertension (47–48). Thus, future interventions targeted at hypertension control need to address the poor hypertension literacy of this population.

Results revealed anxiety and fear of hypertension as important psychosocial barriers to SMBP. Previous qualitative studies revealed conflicting results on SMBP-associated anxiety. In a study on SMBP among pregnant women, the researchers found that SMBP helped to manage the participants’ anxiety regarding their health during pregnancy [49]. However, a qualitative study with stroke patients yielded similar results to our study; investigators found that participants who saw high blood pressure measurements became anxious about their hypertension [50]. Unlike what has been previously documented, our study demonstrated that anxiety and fear of hypertension can inhibit SMBP behaviors and lead to compulsive SMBP behaviors. This data is consistent with the growing literature on mental health disease burden among refugees. For example, several studies have found high rates of anxiety, excessive worry, and obsessive-compulsive disorders among Syrian and Iraqi refugees and asylum seekers [51–56]. Although it is not possible to holistically understand the participants’ mental health based on this study’s data, future research should examine the interactions of health anxiety and self-management of hypertension in this population.

In contrast to participants that are extremely worried about their hypertension, a significant proportion of participants (16.5% for all and 37.5% for Syrians) did not believe/did not know they have hypertension despite having a diagnosis from their providers (Table 2). This lack of disease awareness has been documented in a previous study which demonstrated that foreign-born participants are more likely to be unaware of their hypertension than US-born participants [57]. This pertinent discrepancy highlights the need for providers to gain an awareness of their patients’ disease understanding to improve care and health outcomes. This gap in refugee patients’ understanding may also reflect the language barriers in healthcare for this population. A study of US-resettled refugees demonstrated a high correlation between health literacy and English proficiency [58]. The effective use of professional interpreter services has been shown to reduce clinically significant errors and increase quality of care [59]. Future research should explore the relationship between language barriers and disease awareness among this population.

Many participants in this study were not interested in engaging in SMBP due to their belief in the inaccuracy of their home monitors. This finding supports previous research that found patients were concerned about the reliability of their home blood pressure devices when asked about SMBP [60]. The concerns of these participants are reasonable since only 15% of the 3000 commercially available blood pressure monitors are validated [61]. Since the cost of home monitors is decreasing and data suggests that reimbursement of home monitors is cost beneficial for insurance companies, there is a great incentive to cover patients’ purchases of home monitors, especially since home monitoring improves management and blood pressure control [22–25]. Meanwhile, it is extremely important that clinicians provide patients with resources on selecting validated blood pressure monitors [62].

Learnings from this study highlight the importance of understanding the culture of minoritized communities in the clinical space. Many participants reported a cultural stigma that presents a barrier to effective SMBP and overall hypertension self-management. Similar findings are also cited in previous literature (63, 64). In an Australian study with Arab migrants and refugees, Shahin et al. found that differences in perception of hypertension varied even between Arab refugees and Arab migrants which led to lower medication adherence among refugees, highlighting the need for providing culturally concordant care to this population [47]. Culturally tailored interventions for chronic illnesses improve health outcomes among ethnic minorities [65]. This data also emphasizes the need for community-level targeted interventions to reduce health-related stigma. Community educational approaches can be effective, especially when combined with other approaches such as contact and skills building [66]. Efforts should be taken to develop a culturally appropriate stigma-reduction intervention for this population.

Many of our participants reported not receiving the proper education or training to engage in effective SMBP. These findings confirm prior studies that found inadequate counseling and education from providers is a significant barrier to SMBP and overall hypertension self-management [67–69]. Providers need to encourage SMBP practices within this population with appropriate support on topics such as purchasing monitors, how to measure blood pressure, interpreting blood pressure readings, and the overall importance of SMBP. Innovative modalities for patient education can be implemented, such as an SMS-facilitated home blood pressure monitoring program [70]. Furthermore, since family members served as facilitators to SMBP, it will be worthwhile to implement a family-centered care/empowerment model for this refugee community as previous studies demonstrated the ability of this model to improve the quality of life of patients with hypertension (71–72).

It is important to investigate facilitators and barriers of SMBP among refugee patients from the providers’ perspectives. Current research shows that providers serving a refugee patient population feel they lack the proper education and tools – especially training on culturally appropriate and trauma-informed care – to support this minoritized group (73–74). A qualitative study on physician perceptions of SMBP revealed that lack of workflow support is a common barrier to SMBP [75]. Future studies need to investigate providers’ perceived barriers and facilitators to SMBP specifically for the refugee population. Additionally, efforts need to be taken to develop trainings for providers to support their care of refugees with chronic diseases such as hypertension.

While this study was not designed to focus on SMBP during a pandemic, it is important to note that data revealed how the COVID-19 pandemic negatively impacted SMBP for participants relying on public monitors. Clinicians, researchers, and leaders discussed similar findings on the exacerbation of blood pressure control inequities by COVID-19 [62]. COVID-19 was also highlighted as a barrier to care-seeking behavior in a scoping review on diabetes self-management during the pandemic [76]. These trends warrant further research into the effect of the COVID-19 pandemic and other disasters (e.g., public health, environmental) on hypertension self-management, especially in vulnerable populations such as the refugee community.

## Strengths and limitations

To the best of the authors’ knowledge, this is the first study to investigate SMBP among the refugee population. The large sample size of 109 participants provides strength to this study. While this research has many strengths, there are a few limitations. There is the possibility of social desirability bias in the data collected from participants. However, participants were consistently encouraged by trained data collectors to share their authentic ideas and experiences in an attempt to reduce this bias. Additionally, there is a possibility of self-selection bias as this study was part of a larger investigation into refugee patients’ barriers to hypertension care and opportunities to improve self-management (IRB #200,063) where participants were provided a free home blood pressure monitor. Since participation was voluntary, it is possible that patients who face more barriers to SMBP may be more motivated to participate to receive a new device. It is also important to recognize that the participant group was limited to refugees in San Diego, CA, a region that may not be representative of refugee experiences in other US resettlement cities. However, the goal of this qualitative study is not to be representative of all refugee experiences; rather, this study seeks to capture and elevate the narratives of the participant population. Further research is needed to comprehensively investigate the diversity of the refugee population.

## Conclusions

The present study examines the barriers and facilitators to SMBP among US-resettled Arab refugees with hypertension in San Diego, CA. The data revealed this refugee population faces many challenges to engaging in effective SMBP practices including poor hypertension literacy and lack of encouragement from providers. This research confirmed prior studies that revealed similar barriers to hypertension self-management, especially among minoritized and medically underserved communities. Improved patient education and interventions to support self-management of hypertension are necessary for this refugee community. Additionally, further research engaging providers is needed to develop care models that foster strong patient-provider collaborations and improve health outcomes for refugee patients with hypertension.

## Data Availability

The datasets used and/or analyzed during the current study are available from the corresponding author on reasonable request.

## List of abbreviations

CA: California
SMBP: Self-measured blood pressure monitoring
US: United States
